# Caring for patients in mental health services during COVID-19 outbreak in China

**DOI:** 10.1186/s12991-020-00317-z

**Published:** 2020-11-22

**Authors:** Ruihua Hou, Limin Yang, Zhen Tang, Teng Chen

**Affiliations:** 1 Department of Psychiatry, Clinical and Experimental Sciences, Faculty of Medicine, University of Southampton, Southampton, UK; 2 Shandong Mental Health Centre, Jinan, Shandong China; 3 Suzhou Guangji Hospital, Suzhou, Jiangsu China; 4 Shandong University Qilu Hospital, Jinan, Shandong China

**Keywords:** COVID-19, Mental health services, Psychiatry, Isolation, Testing

## Abstract

This article reflects on some radical changes made in mental health services in China which include the implementation of the initial triage system and the special isolation ward, the early screening and testing for both patients and staff, the smaller teams working on rotating shifts on-site, and the adequate provision of PPE. These measures would be of great value as a reference to the effective delivery of mental health services in other countries through this pandemic.

## Background

The World Health Organization has declared COVID-19 a “public health emergency” [1]. With 173 million people living with mental disorders in China [2], the COVID-19 outbreak has posed an emerging challenge for mental health services in China [3].

Following the report of a cluster of 50 cases of COVID-19 amongst inpatients in one psychiatric hospital in Wuhan, China, on 9th February, 2020 [4], significant concerns were raised. A statement addressed the prevention and control of COVID-19 in patients with severe mental disorders on 17th February, 2020 [5]. Mental health institutions have since taken a series of mandatory measures to address prevention of nosocomial cross-infection between patients and medical staff during the pandemic period, as well as easing difficulties of access to mental health services.

## Main text

### On-site triage

On-site COVID-19 screening sites have been temporarily set up at entrances of hospitals. This includes using infra-red thermometers to screen for fever in all patients and carers, as well as recording travel and contact histories within known epidemic areas over the past month. Patients are issued with coloured cards to label their potential risk of infection (green indicates low risk; yellow indicates moderate risk; red indicates high risk). Essential personal protective measures are required (such as wearing a mask) at this stage.

### Admission pathways

**Fig. 1:**
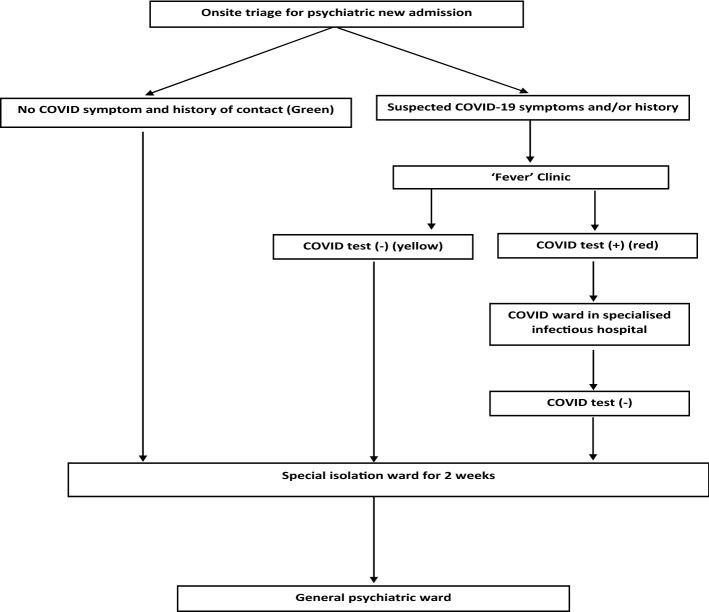
Flowchart for new hospital admission pathways. For all patients requiring hospital admissions, those with no fever or cough or contact and travel history to the epidemic region will go onto isolation wards for 2 weeks for observation. For those with suspected COVID-19 symptoms, they are referred to the temporarily set up site for COVID-19 screening, known as the ‘fever’ clinic run by community physicians, where COVID-19 virus nucleic acid tests are carried out, as well as temperature, blood test, and computed tomography scans of the lungs are taken. For those who have tested positive, transfers to COVID-19 wards in an infectious hospital will be requested immediately. After 2 weeks of observations, a negative test for COVID-19 is then required before admission to the non-infected general wards

Hospitals set up special isolation wards for all new admissions to monitor any COVID-19 symptoms due to the incubation period. After 2 weeks of observations, a negative test for COVID-19 is then required before admission to the non-infected general wards. Stricter measures are introduced to geriatric wards including separation from the rest of the hospital to protect the most vulnerable patients. This pathway is outlined in the flowchart in Fig. 1.

### Medical staff

All medical staff have weekly COVID-19 virus nucleic acid tests taken with routine daily monitoring of body temperature. In special isolation wards, the workforce is divided into smaller subunits to limit the transmission of the virus within teams. The team for each isolation ward is divided into two subgroups with each group working alternate fortnights on the ward; this alternating rota of 14 days work followed by 14 days rest is used for all staff working on the special isolation wards across mental health services. During those 14 working days, all medical staff are accommodated on-site in the hospital, followed by self-isolation for 7 days at their own home. All communication plans are delivered via WeChat online.

### Personal protective equipment (PPE)

Appropriate PPE for all psychiatric staff are provided to minimise transmission via droplets as well as via the faecal–oral route. Due to the increased risk in outpatient clinical settings, all psychiatrists and nurses wear PPE including surgical face masks, long-sleeved disposable gowns, disposable gloves, disposable overshoes, and eye protection goggles. Staff working in isolation wards wear surgical face masks or N95 respirators and long-sleeved disposable gowns. Strict deep-cleaning measures were introduced in both wards and outpatient clinics.

### Online services

To limit the number of outpatient visits, a switch of psychiatric care to online services has been made available. 24/7 hotline is offered to promote wellness by focusing on coping strategies using psychoeducation and cognitive behavioural techniques following the principles for emergency psychological crisis intervention for COVID-19 pneumonia epidemic [6]. In addition, telemedicine is offered to patients requiring referral for mental health evaluation and care, along with increased prescription period from 1 month to 2 months. New treatment advice for COVID-19-related psychiatric symptoms are accessible online [7]. This has substantially reduced the patient flow in the outpatient clinical settings. Outpatient clinics remain open to those patients who are unable to access online services, those who need close monitoring of their physical conditions, and those who need urgent care, with stricter protection measures in place in these services including PPE, deep cleaning, body temperature monitoring, and distancing.

## Conclusions

The key features of the changes to mental health services in China in response to COVID-19 include the implementation of the initial triage system and the isolation ward, the early screening and testing for both patients and staff, the smaller teams working on rotating shifts on-site, and the adequate provision of PPE. As a result of such radical and strict prevention and control measures, the spread of COVID-19 has been contained within the mental health services. However, cases outside of China have been growing rapidly, particularly in the USA and Europe. Under continued spread of COVID-19 worldwide, active and effective measures are critical to ensure the safety, care, and treatment of people affected by mental illness. Measures taken in China should be of great value as a reference to the effective delivery of mental health services in other countries through this pandemic.

## Data Availability

Not applicable.

## Abbreviations

PPE: Personal protective equipment
COVID-19: Coronavirus Disease 2019
